# Attitudes and precaution practices towards COVID-19 among pregnant women in Singapore: a cross-sectional survey

**DOI:** 10.1186/s12884-020-03378-w

**Published:** 2020-11-10

**Authors:** Ryan Wai Kheong Lee, See Ling Loy, Liying Yang, Jerry Kok Yen Chan, Lay Kok Tan

**Affiliations:** 1 Department of Maternal-Fetal Medicine, KK Women and Children’s Hospital, 100 Bukit Timah Road, Singapore, 229899 Singapore; 2 Department of Reproductive Medicine, KK Women and Children’s Hospital, 100 Bukit Timah Road, Singapore, 229899 Singapore; 3 Duke-NUS Medical School, Singapore, 169857 Singapore; 4 Singapore Institute for Clinical Sciences, Agency for Science, Technology and Research (A*STAR), Singapore, 117609 Singapore; 5 Department of Obstetrics and Gynaecology, Singapore General Hospital, Outram Road, Singapore, 169608 Singapore

**Keywords:** Attitudes, Precaution, Practices, COVID-19, Pregnant, Survey

## Abstract

**Background:**

COVID-19 may predispose pregnant women to higher risks of severe disease and poorer neonatal outcome. Psychological sequalae of this pandemic may pose a greater conundrum than its clinical aspects. It is currently unknown that how pregnant women cope with this global pandemic and its ramifications. The aims of the study are to understand the attitudes and precaution practices of non-infected pregnant women towards the COVID-19 outbreak in Singapore.

**Methods:**

An online cross-sectional survey of COVID-19 awareness among pregnant women attending antenatal clinics in Singapore was conducted. An internet link was provided to complete an online electronic survey on Google platform using a quick response (QR) code on mobile devices. The online survey consists of 34 questions that were categorized into 4 main sections, namely 1) social demographics 2) attitude on safe distancing measures 3) precaution practices and 4) perceptions of COVID-19. Multiple linear regression analysis was performed to examine women’s precaution practices among six independent socio-demographic variables, including age, ethnicity, education, front-line jobs, history of miscarriage and type of antenatal clinic (general, high risk).

**Results:**

A total of 167 survey responses were obtained over 8 weeks from April to June 2020. The majority of women were aged ≤35 years (76%, *n* = 127), were of Chinese ethnicity (55%, *n* = 91), attained tertiary education (62%, *n* = 104) and were not working as frontline staff (70%). Using multiple linear regression models, Malay ethnicity (vs. Chinese, β 0.24; 95% CI 0.04, 0.44) was associated with higher frequency of practicing social distancing. Malay women (β 0.48; 95% CI 0.16, 0.80) and those who worked as frontline staff (β 0.28; 95% CI 0.01, 0.56) sanitized their hands at higher frequencies. Age of ≥36 years (vs. ≤30 years, β 0.24; 95% CI 0.01, 0.46), Malay (vs. Chinese, β 0.27; 95% CI 0.06, 0.48) and Indian ethnicity (vs. Chinese, β 0.41; 95% CI 0.02, 0.80), and attendance at high-risk clinic (vs. general clinic, β 0.20; 95% CI 0.01, 0.39) were associated with higher frequency of staying-at-home.

**Conclusion:**

Social demographical factors including age > 36 years old, Malay ethnicity, employment in front line jobs and attendance at high-risk clinics are likely to influence the attitudes and precaution practices among pregnant women towards COVID-19 in Singapore. Knowledge gained from our cross-sectional online survey can better guide clinicians to communicate better with pregnant women. Hence, it is important for clinicians to render appropriate counselling and focused clarification on the effect of COVID-19 among pregnant women for psychological support and mental well being.

**Supplementary Information:**

The online version contains supplementary material available at 10.1186/s12884-020-03378-w.

## Background

Coronavirus disease (COVID-19) is an infectious disease caused by a newly discovered severe acute respiratory syndrome coronavirus (SARS-CoV-2) first identified in Wuhan City, China, in December 2019 [1]. On 11th March 2020, the World Health Organization (WHO) declared the COVID-19 outbreak as a global pandemic with exponential spread worldwide [2]. As of 4 August 2020, there are currently over 18 million people globally affected by COVID-19 with over 700,000 deaths reported worldwide, and rising [3]. Since the first case of COVID-19 was confirmed in Singapore on 23 January 2020, local transmission began to develop in February and March 2020 with soaring numbers averaging 400–500 new cases of COVID-19 per day from April to June 2020 [4, 5]. Consequently, the Singapore government introduced safe distancing measures also known as ‘circuit-breaker’ to pre-empt the trend of increasing transmission of COVID-19 by reducing significantly movements and interactions in places from 7 April 2020 till 1 June 2020 [6]. Since then, the total confirmed cases are over 53,000 with 27 deaths based on the Ministry of Health’s (MOH) report on 4 August 2020) [7].

The effects of SARS-CoV-2 in pregnancy were initially based upon previous experience with SARS-CoV-1 and Middle East Respiratory Syndrome-related coronavirus (MERS) [8, 9]. However, SARS-CoV-2 turns out to be far more infectious, albeit with lower mortality and similar morbidity to women of reproductive age [10]. The rapidly evolving pandemic over the past 6 months has given rise to multiple living-guidelines for the management of COVID-19 in pregnancy from a range of professional bodies such as the Royal College of Obstetricians & Gynaecologists (RCOG), American College of Obstetricians and Gynaecologists (ACOG) and the Academy of Medicine in Singapore [11–13]. As our knowledge of COVID-19 increases, hospital recommendations on infection control, COVID-19 screening and isolation protocols change rapidly in accordance with the latest evidence.

The physiological and immunological changes in pregnancy make women more susceptible to severe illness from respiratory infections [14, 15]. A recent Centre for Disease Control and Prevention (CDC) report demonstrated that pregnant women with COVID-19 are more likely to be hospitalised, admitted to the intensive care unit and receive mechanical ventilation albeit with similar risk of mortality compared to non-pregnant women [16].

Pregnancy itself poses logistical challenges and conundrums for obstetricians managing pregnant women with suspected or diagnosed with COVID-19. The RCOG suggests that the COVID-19 pandemic increases the risk of perinatal anxiety, depression, and domestic violence in pregnant women [11]. Hence, pregnant women deserve a more sensitive approach and mutual understanding during this global pandemic among clinicians and their partners. There are limited studies assessing the attitude and public perceptions towards the effect of COVID-19 among pregnant women [17, 18]. As the COVID-19 pandemic continues to intensify globally, it is important to understand the mentality of pregnant women towards COVID-19. Consequently, this will enable clinicians to provide appropriate counselling to reassure and clarify doubts of pregnant women towards COVID-19 during the antenatal, intra partum and post- partum period.

Social media and information access in Singapore are readily available via the internet with majority owning mobile devices. Thus, establishing public awareness of COVID-19 using an online survey is easily achieved in a developed country like Singapore using information technology for disseminating and receiving information on social media. We reported the results from a rapid online cross-sectional survey related to COVID-19 among pregnant women attending antenatal clinics in Singapore. The survey aimed to 1) establish the baseline attitudes of pregnant women towards COVID-19 and 2) correlate socio-demographics with women’s precaution practices towards COVID-19 in Singapore. This online survey will help identify various characteristics of pregnant women who are more likely to be vulnerable towards the effects of COVID and enable clinicians to reflect on the insecurities and worries of pregnant women for more focused counselling.

## Methods

We conducted an online cross-sectional survey for pregnant women attending antenatal clinics in two large tertiary-referral hospitals in Singapore, namely KK Womens’ Childrens’ Hospital and Singapore General Hospital from April to June 2020. These two hospital have approximately twelve-thousand and two-thousand pregnancies and deliveries respectively per year, accounting for more than 50% of the total number of pregnancies and deliveries per year in Singapore [19, 20]. Ethics approval for the study including waiver of informed consent was obtained from the Singhealth Centralised Institutional Review Board (CIRB 2020/2307).

Pregnant women attending antenatal clinics were provided with an internet link to complete an online electronic survey on Google platform using a quick response (QR) code on any mobile device with internet access. The survey was anonymous and could be completed in about 10 min. The online electronic survey was created using CHERRIES (Checklist for Reporting Results of Internet E-Surveys) [14] and the questions were designed by a group of senior obstetricians.

The online survey consisted of 34 questions that were categorized into 4 main sections, namely 1) social demographics (Q1-Q10), 2) attitude on safe distancing measures (Q11–17), 3) precaution practices towards COVID-19 (Q18–21) and 4) perceptions of COVID-19 in the antepartum period (Q22-Q27), intra-partum care (Q28-Q30) and post-partum care. (Q31-Q34). (Additional file 1).

The survey was designed to capture general awareness of COVID-19 and perceived views on COVID-19 including social distancing measures, preferred mode of delivery, willingness to separate from their child at birth and avoiding breast feeding to minimize the risk of vertical neonatal transmission. ‘High risk’ pregnant women with obstetric indications attended high-risk clinics whereas ‘low risk’ pregnant women attended general clinics.

Responses to the questions were rated in different scales, 1) Yes, No, Not Sure, or 2) Not often, Occasionally, Often, Very often, or 3) Never, Rarely, Sometimes, Usually, Always. The different types of response scales were determined based on the forms and appropriateness of questions asked. Respondents did not receive any incentive to complete the survey and standard of care was not affected if they did not participate in the online survey. Respondents had to provide a response to every question to complete the survey. The electronic data were compiled and saved on a secured website that was password protected to access the data with no identifiable patient information available.

Women’s characteristics and distributions of their attitudes, practices and perceptions towards COVID-19 were presented in frequencies and percentages. Multiple linear regression analysis was performed to examine the main factors associated with women’s precaution practices among the six-independent socio-demographic variables, including age (≤30, 31–35, ≥36 years), ethnicity (Chinese, Malay, Indian, others), education (primary or secondary, post-secondary, tertiary), front-line jobs (no, yes), history of miscarriage (no, yes) and type of antenatal clinic (general, high risk). The scales of the dependent variables were treated in continuous form based on the design of rating in a continuum sequence of values, which could also help to increase the power of analysis. Data were presented as β coefficients and 95% confidence interval (CIs). Statistical analysis was performed using the IBM SPSS Statistic Package, version 20.0 (IBM Corp., Armonk, N.Y., USA).

## Results

A total of 167 survey responses were obtained over 8 weeks from April to June 2020. The clinical characteristics and demographics are presented in Table 1. Among the included women, the majority of them were aged ≤35 years (76%, *n* = 127), were of Chinese ethnicity (55%, *n* = 91), attained tertiary education (62%, *n* = 104) and were not working as frontline staff (70%). This was representative of the social-demographics in the Singapore study population. In terms of obstetric history, most women conceived naturally (90%, *n* = 149), were primiparous (51%, *n* = 85) and at their third trimester of pregnancy (44%, *n* = 74), had no history of miscarriage (80%, *n* = 134) and were currently followed up in general clinics (75%, *n* = 125).

**Table 1:** Characteristics of participants (*n* = 167)

Demographics	n (%)
Age	
≤ 30 years	56 (33.5)
31–35 years	71 (42.5)
≥ 36 years	40 (24.0)
Ethnicity	
Chinese	91 (54.5)
Malay	50 (29.9)
Indian	8 (4.8)
Others	18 (10.8)
Religion	
Buddhist	34 (20.4)
Christian	28 (16.8)
Islam	59 (35.3)
Others	46 (27.5)
Education	
Primary/ secondary	16 (9.6)
Post-secondary	47 (28.1)
Tertiary	104 (62.3)
Frontline job	
No	116 (69.5)
Yes	51 (30.5)
Type of conception	
Natural	149 (89.8)
IVF/IUI	17 (10.2)
Trimester	
First < 13 weeks’ gestation	30 (18.0)
Second 13–26 weeks’ gestation	63 (37.7)
Third 27–40 weeks’ gestation	74 (44.3)
Number of living children	
0	85 (50.9)
1	55 (32.9)
≥ 2	27 (16.2)
History of miscarriage	
No	134 (80.2)
Yes	33 (19.8)
Type of clinic	
General	125 (74.9)
High-risk	42 (25.1)

Table 2 and Table 3 shows the distribution of participants’ attitude (Q11–17), precaution practices (Q18–21) and perceptions (Q22–34) towards COVID-19 in pregnancy. One hundred twenty-four women (74%) were worried and very worried about being infected with COVID-19 in pregnancy (Q23). Seventy-seven (46%) women were unsure if pregnant women infected with COVID-19 are more likely to miscarry or go into pre-term labour (Q27). Seventy-eight (47%) women think that there is high risk of COVID-19 infection to their baby at the time of delivery if they were diagnosed with COVID-19 (Q25) and eighty-nine (53%) women would choose having a caesarean section over a vaginal delivery if they were diagnosed with COVID-19 (Q30). After delivery, fifty-eight (35%) women preferred to breast feed if they were diagnosed with COVID-19 (Q34). These questions did not show any association in relation to socio-demographic factors (data not shown).

**Table 2:** Distribution of participants attitude (Q11–17) and precautions (Q18–21) during towards COVID-19

Attitude	Not often	Occasionally	Often	Very often	
	n (%)	n (%)	n (%)	n (%)	
Q11 How often do you check for COVID-19 related news on social media?	1 (0.6)	16 (9.6)	59 (35.5)	90 (54.2)	
	**No**	**Not sure**	**Yes**		
Q12 Have you had any COVID-19 swab test done before for suspected COVID-19?	159 (96.4)	0	6 (3.6)		
Q13 Do you know of any close family/extended family members diagnosed with COVID-19?	164 (98.2)	3 (1.8)	0		
Q14 Have you received any official stay home notice/home quarantine order in this current pregnancy?	161 (96.4)	0	6 (3.6)		
Q15 Do you know of any close family/extended family members who have been issued with a stay home notice/home quarantine order?	145 (86.8)	0	22 (13.2)		
Q17 Have you missed any clinic appointments because of the fear of being infected with COVID-19?	1 59 (95.2)	0	8 (4.8)		
	**Not important**	**Not so important**	**Neutral**	**Somewhat important**	
Q16 If you are well, how important do you think it is to come for your antenatal appointments?	1 (0.6)	3 (1.8)	13 (7.8)	24 (14.4)	
**Precautions**	**Never**	**Rarely**	**Sometimes**	**Usually**	**Always**
	**n (%)**	**n (%)**	**n (%)**	**n (%)**	**n (%)**
Q18 How often do you practise social distancing in this current pandemic?	1 (0.6)	0	2 (1.2)	28 (16.9)	135 (81.3)
Q19 How often do you stay home for social distancing?	0	0	8 (4.8)	45 (26.9)	114 (68.3)
Q20 How often do you wear a mask at home?	124 (74.3)	33 (19.8)	6 (3.6)	0	4 (2.4)
Q21 How often do you sanitize your hands using hand-rub or hand-wash?	0	5 (3.0)	26 (15.6)	52 (31.1)	84 (50.3)

**Table 3:** Distribution of participants’ perceptions (Q22–34) towards COVID-19

Antepartum	Not often	Occasionally	Often	Very often	
	n (%)	n (%)	n (%)	n (%)	n (%)
Q22 Do you think that pregnant women will be at higher risk of getting severe respiratory illness compared to non-pregnant women?	31 (18.6)	51 (30.5)	85 (50.9)	0 (0)	
	**Not worried**	**Not sure**	**Neutral**	**Worried**	**Very worried**
Q23 How worried are you about being infected with COVID-19 during your pregnancy?	4 (2.4)	1 (0.6)	38 (22.8)	74 (44.3)	50 (29.9)
	**Low**	**Medium**	**High**	**Unsure**	
Q24 If you are diagnosed have COVID-19, what you do think is the risk of infection to the baby **before** delivery	23 (13.8)	35 (21.0)	61 (36.5)	48 (28.7)	
Q25 If you are diagnosed have COVID-19, how likely do you think is the risk of infection to the baby **during** delivery	11 (6.6)	36 (21.6)	78 (46.7)	42 (25.1)	
Q26 If you are diagnosed have COVID-19, how likely do you think is the risk of infection to the baby **after** delivery	6 (3.6)	29 (17.4)	90 (53.9)	41 (24.6)	
	**Very unlikely**	**Unlikely**	**Neutral**	**Likely**	**Very likely**
Q27 Do you think pregnant women infected with COVID-19 are more likely to miscarry or go into labour early?	5 (3.0)	30 (18.0)	77 (46.1)	41 (24.6)	14 (8.4)
	**No**	**Not sure**	**Yes**		
**Intrapartum**					
Q28 As there may be infection risks during your time of delivery, will you agree to have an epidural for pain relief during your time of delivery if you are **suspected/diagnosed** with COVID-19?	20 (12.0)	52 (31.1)	95 (56.9)		
Q29 As we do not know enough about the risk of transmission of the COVID-19 infection to your baby, this may influence or affect the mode of delivery, hence will you agree if your doctor will advised you for caesarean section over a vaginal delivery if you are **suspected** to have COVID-19?	35 (21.0)	59 (35.3)	73 (43.7)		
Q30 As we do not know enough about the risk of transmission of the COVID-19 infection to your baby, this may influence or affect the mode of delivery, hence will you agree if your doctor will advised you for caesarean section over a vaginal delivery if you are **diagnosed** with COVID-19?	21 (12.6)	57 (34.1)	89 (53.3)		
**Postpartum**					
Q31 Do you think it is safe for infected women to have close contact (skin to skin) with their baby after delivery?	96 (57.5)	33 (19.8)	38 (22.8)		
Q32 Will you isolate away from your baby for 2 weeks after delivery if you are infected with COVID-19	22 (13.2)	33 (19.8)	112 (67.1)		
Q33 Under normal conditions (with no COVID-19), would you breastfeed your baby?	7 (4.2)	4 (2.4)	156 (93.4)		
Q34 If you were infected, with COVID-19, would you still breastfeed your baby?	66 (39.8)	42 (25.3)	58 (34.9)		

Table 4 shows the associations of women’s socio-demographics with precaution practices towards COVID-19 based on multiple linear regression models. Malay (vs. Chinese, β 0.24; 95% CI 0.04, 0.44) was associated with higher frequency of practicing social distancing (Q18). Age of ≥36 years (vs. ≤30 years, 0.24; 0.01, 0.46), Malay (vs. Chinese, 0.27; 0.06, 0.48) and Indian ethnicity (vs. Chinese, 0.41; 0.02, 0.80), and attendance to high-risk clinic (vs. general clinic, 0.20; 0.01, 0.39) were associated with higher frequency of staying-at-home behaviour (Q19); whereas front-line job (vs. non-front-liner, − 0.22; − 0.40, − 0.04) and miscarriage experience (vs. no history of miscarriage, − 0.28; − 0.49, − 0.07) were associated with lower frequency of home staying. Compared to women aged ≤30 years, those aged 31–35 years (− 0.33; − 0.61, − 0.05) and ≥ 36 years (− 0.36; − 0.69, − 0.04) were less often to wear masks at home (Q20). In terms of hand hygiene practices, Malay women (0.48, 0.16, 0.80) and those who worked as frontline staff (0.28, 0.01, 0.56) sanitized their hands at higher frequencies (Q21).

**Table 4:** Characteristics associated with precaution practices towards COVID-19

Demographics	Q18	Q19	Q20	Q21
Questions	How often was social distancing practiced	How often did they stay home	How often was a mask worn at home	How often was hand hygiene practiced
	β (95% CI)	β (95% CI)	β (95% CI)	β (95% CI)
Age				
≤ 30 years	1.00	1.00	1.00	1.00
31–35 years	0.02 (−0.17, 0.21)	0.10 (−0.10, 0.29)	−0.33 (−0.61, −0.05)	−0.02 (−0.32, 0.29)
≥ 36 years	0.21 (−0.01, 0.43)	0.24 (0.01, 0.46)	−0.36 (− 0.69, − 0.04)	0.29 (− 0.06, 0.63)
Ethnicity				
Chinese	1.00	1.00	1.00	1.00
Malay	0.24 (0.04, 0.44)	0.27 (0.06, 0.48)	0.02 (−0.29, 0.32)	0.48 (0.16, 0.80)
Indian	0.31 (−0.06, 0.69)	0.41 (0.02, 0.80)	0.02 (−0.55, 0.58)	0.37 (−0.24, 0.97)
Others	−0.02 (− 0.39, 0.25)	−0.16 (− 0.44, 0.12)	−0.28 (− 0.68, 0.12)	0.19 (− 0.24, 0.61)
Education				
Primary/ secondary	1.00	1.00	1.00	1.00
Post-secondary	0.04 (−0.26, 0.34)	−0.08 (− 0.40, 0.24)	0.11 (− 0.35, 0.56)	−0.14 (− 0.62, 0.34)
Tertiary	0.23 (− 0.07, 0.52)	−0.23 (− 0.53, 0.08)	−0.06 (− 0.50, 0.38)	−0.12 (− 0.59, 0.35)
Frontline job				
No	1.00	1.00	1.00	1.00
Yes	−0.12 (− 0.29, 0.06)	−0.22 (− 0.40, − 0.04)	−0.03 (− 0.28, 0.23)	0.28 (0.01, 0.56)
History of miscarriage				
No	1.00	1.00	1.00	1.00
Yes	−0.12 (−0.32, 0.08)	− 0.28 (− 0.49, − 0.07)	−0.11 (− 0.42, 0.19)	−0.32 (− 0.64, 0.01)
Type of clinic				
General	1.00	1.00	1.00	1.00
High-risk	0.08 (−0.10, 0.27)	0.20 (0.01, 0.39)	−0.03 (− 0.30, 0.25)	−0.02 (− 0.31, 0.27)

Data were analysed using the multiple linear regression models. *CI* confidence interval

## Discussion

To the best of our knowledge, our study is hitherto the first study performed in a South East Asian population of pregnant women. Factors like race, religion, education background and employment status can influence women’s attitude, practice and perception especially in an affluent country like Singapore. Our survey showed that Malay pregnant women are likely to practice safe distancing and sanitise their hands at a higher frequency compared to Chinese to minimise the spread of COVID-19. In addition, women attending ‘high-risk’ clinics are more likely to stay at home compared to women attending ‘general clinics’. A plausible explanation suggests that women with high risk pregnancies are more likely to stay at home to minimize themselves or their foetuses from being infected with COVID-19 when compared to women with low risk pregnancies.

Employed individuals who worked in front line services such as healthcare, hospitality have a lower tendency to stay home for social distancing, possibly driven by their more sociable or out-going characteristics when compared to those do not work in front line. Conversely, our study also showed that employed individuals with front line jobs are more likely to practice hand hygiene compared to those who do not to reduce the risk of infection. It is possible to attribute this to the nature characteristics of front-line jobs and these women are likely to have a high tendency to wash their hands more often compared to those who do not work in front-line jobs. In our study, women with previous history of miscarriage had a lower tendency to stay home for maintaining social distancing (Q19, β: − 0.22) suggesting that obstetric experience did not make women more cautious to practice social distancing to protect themselves. The same inverse associations were observed for Q18,Q20, Q21 with no significance.

There are currently limited cross-sectional studies addressing the attitude and perception of COVID-19 among pregnant women. Anikwe et al. showed that majority of pregnant women in their third trimester in Nigeria demonstrated good attitude and preventative practices of COVID-19 [21] by practising hand washing, wearing masks, avoiding face touching and quarantine infected people as good practices towards the prevention of COVID-19 infection. These measures were performed without a ‘lock-down’ period unlike Singapore which implemented a colour-coded framework known as ‘Disease Outbreak Response System Condition’ (DORSCON) to guide the public on prevention and reducing the impact of COVID-19. There are four statuses namely Green, Yellow, Orange and Red of which Singapore is at orange currently which meant that the disease is severe but has not spread widely and is being contained [22]. The Singapore government implemented a ‘circuit-breaker’ in different phases’ akin to lock-down period in other countries to curb the community spread of COVID-19 [23]. A circuit breaker is a set of safe distancing measures akin to a lock-down to pre-empt the trend of increasing transmission of COVID-19 by reducing significantly movements and interactions in places [6]. Safety measures implemented include staying mostly indoors and going outdoors only when necessary, practice social distancing at least one metre apart, wearing surgical masks in public places and adopting good hand sanitation practices to reduce the risk of community spread of COVID-19. Singaporeans are mostly compliant to the safe distancing measures as there are strict rules regarding social distancing with hefty fines and custodial sentences along with an effective enforcement ability.

Pregnant women should be appropriately educated on preventative measures to reduce the severity of COVID-19 associated illness. Pregnant women should also avoid missing prenatal appointments if well and limit interactions with others to reduce the risk of transmission. Symptomatic women should be urged to be tested early for COVID-19 by nasopharyngeal or oropharyngeal swabs and practice self-isolation to reduce the risk of vertical transmission [24, 25].

Yassa et al. focused on Turkish pregnant women in attitude, concerns and knowledge towards COVID-19 from 30 weeks gestation onwards [26] where Turkey was one of the most affected countries then with over 20,000 cases and 425 deaths in April 2020 [27]. They showed that about 80% of women felt vulnerable towards the outbreak 45% of women were confused or doubtful about the mode of delivery and 50% wasn’t sure if breast feeding was safe during the pandemic [26]. This is similar to our findings where 74% of women were worried about being infected with COVID-19; 53% of women would choose having a caesarean section over a vaginal delivery and only 35% of women will choose to breast feed if they were diagnosed with COVID-19. These views reflect the vulnerability of pregnant women despite differences in race or culture as pregnant women want the best outcome for themselves and minimize risk of vertical transmission to their baby. Hence it is paramount for clinicians to reflect on the insecurities and worries of pregnant women towards COVID-19.

In our study, 46% of pregnant women believed they are more likely to go into pre-term labour when infected with COVID-19. Di Mascio et al. showed that 41.1% of pregnant women with COVID-19 had preterm birth before 37 weeks gestation, however that study did not distinguish between spontaneous and iatrogenic preterm birth [28]. A systemic review by A. Khalil et al. also showed an 18.4% increase in iatrogenic preterm births before 37 weeks as these women were ill enough to require early caesarean deliveries [10]. This emphasizes the importance of imparting knowledge and educating women to avoid unnecessary anxieties from non-evidenced based perceptions.

In our study, 46% of pregnant women also believed they are more likely to miscarry when infected with COVID-19. A systematic review by Zaigham et al. did not report any adverse outcomes relating to perinatal outcomes [29]. Although results from the SARS epidemic did not suggest an increased risk of miscarriage or congenital anomalies associated with COVID-19 infection, more data is required before conclusions can be made on the risk of miscarriage [30].

In our study, almost three in four (74%) of women were worried and very worried about being infected with COVID-19 in pregnancy. Durankus et al. showed that pregnant women scored higher on the Edinburgh Postpartum Depression Scale (EPDS) when compared to the control group [31]. It is understandable for pregnant women to be anxious and this can be associated with a higher risk of depression [32]. This highlights the importance of providing psychosocial support especially in a vulnerable group of pregnant women. Clinicians should work in tandem with clinical psychologists and psychiatrists in a multi-disciplinary setting. The care of pregnant women should be tailored individually for the mental health of women and their babies.

Most cases of COVID-19 have evidence of human-to-human transmission where the virus appears to spread through respiratory, fomite or faecal methods [33, 34]. There is also emerging opinion that the fetus may be exposed during pregnancy. Perinatal infection may occur but its true incidence remains unknown. The likelihood of vertical transmission is low based on the United Kingdom Obstetric Surveillance System (UKOSS) interim study where six babies (2.5%) had a positive nasopharyngeal swab for SARS-CoV-2 within 12 h of birth in severely affected hospitalised women [35]. Hence, the risk of vertical transmission in mild or asymptomatic patients is likely to be lower than that.

A case series published by Chen et al. a tested amniotic fluid, cord blood, neonatal throat swabs and breast milk samples from COVID-19 infected mothers and all samples tested negative for the virus [36]. Conversely, two reported cases of possible vertical transmission showed evidence of immunoglobulin M (IgM) for SARS-CoV-2 in the neonatal serum [37, 38]. Although direct evidence of viral positive reverse transcriptase-polymerase chain reaction (RT-PCR) were mostly negative in large majority of reported studies, the paucity of published data is limited with small cohort numbers, limited sensitivity and specificity of swab tests and rapid evolution of COVID-19 infection [39–42].. Hence, more data is needed about the risk of vertical transmission before definitive conclusions can be made.

The mode of delivery should be discussed adequately with pregnant women taking into consideration their preferences and any obstetric indications. In our study, 53% of women would choose to have a caesarean section over a vaginal delivery if they were diagnosed with COVID-19. A. Khalil et al. showed that nearly half of pregnant women infected with COVID-19 had caesarean deliveries [10]. As there is no convincing evidence of vertical transmission, vaginal delivery is not contraindicated in patients with COVID-19 [11, 12]. Thus, Caesarean section is preferred over vaginal delivery in the face of maternal deterioration and fetal compromise where delivery is imminent. However, logistical issues can arise from the transfer of patients in hospital to labour ward or the availability of operating theatre to perform a caesarean section with negative pressure to minimize the risk of transmission. Hence, clinicians should counsel women on the appropriate mode of delivery as there is a lack of data and uncertainty surrounding the risk of perinatal transmission during vaginal deliveries.

In our study, only 35% of pregnant women will choose to breast feed if they were diagnosed with COVID-19. There is also limited data to guide the postnatal management of babies of mothers who tested positive for COVID-19 in the third trimester of pregnancy. Currently, possibility of infection from breast milk remain uncertain although there is recent evidence to suggest a small risk of transmission through breast feeding [43–45]. As breast feeding requires close contact, direct breast feeding may be of concern in infected mothers. Hence, infected mothers should be advised to wear surgical masks, cleaning their breast before expression via breast pumps to bottle feed their neonates to reduce the risk of neonatal transmission. Precautionary separation of mother and child is debatable and cause loss of physical bonding and emotional attachment which have a negative psychological impact in infected women.

We chose to perform an online survey as this is a rapid and convenient mode of administration. Furthermore, we used CHERRIES to ensure the quality of our web-based survey [46]. Limitations of our study include small sample size and lack of internal consistency of questions without validation. A larger study would be essential to confirm our findings. Despite our small sample size, the data collected likely representative of our local population as the two large public hospitals which make up more than half of the number of pregnancies and deliveries in Singapore. In addition, our findings may be influenced by possible selection bias because participants needed a mobile device with applications to scan the QR code to access the survey. However, the large majority of the local Singaporean population own mobile devices which makes the online survey easily accessible for participation.

Ever-since the WHO declared COVID-19 a global pandemic, the world has seen an exponential number of rising cases and unprecedented death rates. Until a vaccine is found, herculean efforts rests on containing community spread of COVID-19 through means like testing for suspected cases, practising social distancing and maintaining good personal hygiene [47–49].

## Conclusion

As much of COVID-19 remains hitherto unknown, current opinions regarding management of COVID-19 positive women may change with input of new knowledge. The physical burden of pregnancy makes it psychologically and emotional challenging in vulnerable pregnant women. Social demographical factors including age > 36 years old, Malay ethnicity, employment in front line jobs and attendance at high-risk clinics are likely to influence the attitude and precaution measures among pregnant women towards COVID-19 in Singapore. Knowledge gained from our cross-sectional online survey can better guide clinicians to communicate better with pregnant women. Our study highlights the importance for clinicians to render appropriate counselling and focused clarification on the effect of COVID-19 among pregnant women for psychological support and mental wellbeing.

## Supplementary Information

**Additional file 1.**

## Data Availability

The datasets used during the current study are available from the corresponding author on reasonable request.

## Abbreviations

ACOG: American College of Obstetricians and Gynaecologists
CDC: Centre for Disease Control and Prevention
CIRB: Centralised Institutional Review Board
CHERRIES: Checklist for Reporting Results of Internet E-Surveys
COVID-19: Coronavirus disease (COVID-19)
DORSCON: Disease Outbreak Response System Condition
EPDS: Edinburgh Postpartum Depression Scale
MOH: Ministry of Health
MERS: Middle East Respiratory Syndrome-related coronavirus
UKOSS: United Kingdom Obstetric Surveillance System
QR: Quick response
RCOG: Royal College of Obstetricians & Gynaecologists
RT-PCR: Reverse transcriptase polymerase chain reaction
SARS-COV-2: Severe acute respiratory syndrome coronavirus
WHO: World Health Organization
