# Supplementary material for: Attitudes and precaution practices towards COVID-19 among pregnant women in Singapore: a cross-sectional survey
Source: BMC Pregnancy Childbirth. 2020 Nov 10;20:675. doi: 10.1186/s12884-020-03378-w (PMC7652671; doi:10.1186/s12884-020-03378-w)
Supplement: Supplementary file 1 — Additional file 1. [file 12884_2020_3378_MOESM1_ESM.docx]

**Appendix 1 Online Survey**

**1.Demographics**

1.How old are you now ? ( < 30, 31-35, >35 years old)

2.What is your race/ethnicity? (Chinese, Malay, Indian, Eurasian /Caucasian, Others)

3.What religious affiliation are you? (Christianity, Islam, Buddhism , Others)

4.What is your highest level of education (Primary, Secondary , Tertiary)

5.Are you currently employed in a frontline role (a job with daily customer interaction e.g. transport, service, medical) ? ( Yes, No )

6.Is your current pregnancy a spontaneous or by assisted reproductive techniques (ART)? ( Spontaneous , ART)

7. Which trimester are you in this pregnancy ( first, second, third)

8. How many living children do you have now ( 0, 1, >2)

9. Do you have any history of miscarriage ? ( Yes, No)

10. Which clinic are you being followed up at in our hospital (General clinic, High risk clinic)

**2. Attitude in the antepartum period**

11.How often do you check for COVID-19 related news on social media? ( Very often, often, occasionally, not often )

12.Have you had any COVID-19 swab test done before for suspected COVID-19 ? ( Yes, No)

13.Do you know of any close family/extended family members diagnosed with COVID-19? ( Yes, no, not sure)

14.Have you received any official stay home notice/ home quarantine order in this current pregnancy ? ( Yes, no)

15.Do you know of any close family/extended family members who have been issued with a stay home notice/home quarantine order ? ( Yes, No, Not sure)

16.If you are well, how important do you think it is to come for your antenatal appointments? ( Very, somewhat, not sure, not important)

17.Have you missed any clinic appointments because of the fear of being infected with COVID-19 ? (Yes, No)

**3.Perceptions in the antepartum period**

18. How often do you practise social distancing in this current pandemic ? (very often, often, sometimes, hardly, never)

19.How often do you stay home for social distancing ? (very often, often, sometimes, hardly, never)

20.How often do you wear a mask at home ? (always, usually, sometimes, rarely, never)

21.How often do you sanitize your hands using hand-rub or hand-wash (Always, usually, sometimes, rarely, never)

22. Do you think that pregnant women will be at higher risk of getting severe respiratory illness compared to non-pregnant women ? (yes , no , not sure)

23. How worried are you about being infected with COVID-19 during your pregnancy? ( very, worried, neutral, not worried, not sure)

24.If you are diagnosed have COVID-19, what you do think is the risk of infection to the baby **before** delivery ( low , medium, high, unsure)

25.If you are diagnosed have COVID-19, how likely do you think is the risk of infection to the baby **during** delivery ( ( low , medium, high, unsure)

26.If you are diagnosed have COVID-19, how likely do you think is the risk of infection to the baby **after** delivery ( (low , medium, high, unsure)

27.Do you think pregnant women infected with COVID-19 are more likely to miscarry or go into labour early? (very likely, likely, neutral, unlikely, very unlikely)

**4.Intra-partum care**

28.As there may be infection risks during your time of delivery, will you agree to have an epidural for pain relief during your time of delivery if you are suspected/diagnosed with COVID-19 ? (Yes, No, Not sure)

29.As we do not know enough about the risk of transmission of the COVID-19 infection to your baby, this may influence or affect the mode of delivery, hence will you agree if your doctor will advised you for caesarean section over a vaginal delivery if you are **suspected** to have COVID-19? (Yes, No, Not sure)

30. As we do not know enough about the risk of transmission of the COVID-19 infection to your baby, this may influence or affect the mode of delivery, hence will you agree if your doctor will advised you for caesarean section over a vaginal delivery if you are **diagnosed** with COVID-19? (Yes, No, Not sure)

**5. Post-partum care**

31.Do you think it is safe for infected women to have close contact ( skin to skin ) with their baby after delivery ? (Yes, No, Not sure)

32 Will you isolate away from your baby for 2 weeks after delivery if you are infected with COVID-19 (Yes, No, Not sure)

33.Under normal conditions (with no COVID-19), would you breastfeed your baby? (Yes, No, Not sure)

34. If you were infected, with COVID-19, would you still breastfeed your baby? (Yes, No, Not sure)
